# Influence of Hydrothermal Carbonization Parameters on Surface Properties and Methylene Blue Adsorption Performance of Rose Stem-Derived Hydrochar

**DOI:** 10.3390/molecules31203758

**Published:** 2026-10-19

**Authors:** Carlos Navas-Cárdenas, Cristian Gil, Javier Sayavedra-Delgado, Clara Isabel Ruiz-Sánchez, Luis Corredor, Erika Murgueitio-Herrera, Wilson Navas-Pinto, Alexis Debut

**Affiliations:** 1 Departamento de Ciencias de la Energía y Mecánica, Universidad de las Fuerzas Armadas ESPE, Sangolquí 171103, Ecuadorjjsayavedra@espe.edu.ec (J.S.-D.); ciruiz4@espe.edu.ec (C.I.R.-S.); wmnavas1@espe.edu.ec (W.N.-P.); 2 Centro de Nanociencia y Nanotecnología, Universidad de las Fuerzas Armadas ESPE, Sangolquí 171103, Ecuador; esmurgueitio@espe.edu.ec (E.M.-H.); apdebut@espe.edu.ec (A.D.); 3 School of Chemical Sciences and Engineering, Yachay Tech University, Hda. San José s/n y Proyecto Yachay, Urcuquí 100119, Ecuador; lcorredor@yachaytech.edu.ec; 4 Departamento de Ciencias de la Tierra y la Construcción, Universidad de las Fuerzas Armadas ESPE, Sangolquí 171103, Ecuador

**Keywords:** hydrochar, *Rosa rubiginosa*, hydrothermal carbonization, methylene blue adsorption, circular economy

## Abstract

This research investigated the effect of the synthesis conditions on the adsorption capacity of hydrochar (HC) derived from *Rosa rubiginosa* stems via hydrothermal carbonization. HC samples were prepared at 180, 200 and 220 °C using masses with three different raw-material-to-water ratios (i.e., 5, 7.5 and 10 g of biomass in 80 mL of water), obtaining yields between 43 and 57%. The point of zero charge (PZC) of all samples was determined, obtaining the highest value (5.36) for the sample prepared at 180 °C using 5 g of biomass: 80 mL water ratio (HC5_180). This sample exhibited a crystallinity index value (I_D_/I_G_, determined by Raman Spectroscopy) of 1.73, which is indicative of a higher degree of structural disorder, related to a greater presence of defects generated by surface functional groups, which, in turn, was identified by infrared spectroscopy. Adsorption tests were conducted using methylene blue (MB), achieving 76.3% removal at an initial concentration of 2.5 mg/L. Adsorption isotherms were also obtained for each HC type, which were fitted to the Langmuir and Freundlich models to elucidate the adsorption mechanism, while kinetic studies were performed on the HC5_180 sample. The findings demonstrate that HC adsorption capacity is governed by the distribution and type of surface functional groups. Thus, the valorization of floral industry by-products into biosorbents, as HC, promotes the circular economy, converting operational waste into a sustainable raw material with added commercial value.

## 1. Introduction

Hydrochar (HC) is a porous carbonaceous material obtained from biomass through a thermochemical process in a low-or no-oxygen environment. This material has gained recognition due to its versatility in various applications, such as soil remediation and water treatment. HC can improve soil fertility, enhancing their water-holding capacity and nutrient availability, in addition to helping reduce acidity and prevent the leaching of contaminants [1]. In water treatment, HC can act as a highly efficient adsorbent, allowing the removal of heavy metals, pesticides and organic compounds. Due to its porous structure and large surface area, it can capture and retain these contaminants, thus contributing to water purification and environmental preservation [2,3]. In addition, hydrothermal carbonization (HTC) is an appropriate process used to thermochemically convert waste biomass into gaseous, liquid and solid products at temperatures between 150 and 300 °C in the presence of water. One of the most important products obtained by HTC from biomass is HC, which is a very important material in environmental applications, particularly in the adsorption of contaminants such as organic dyes [4,5]. These dyes, widely used in the textile industry, present environmental challenges due to their resistance to natural degradation [6].

Previous studies reported that the types of HC obtained by HTC of organic waste can have a great capacity to adsorb contaminants [7]. For example, HC derived from olive waste has been used to treat water contaminated with triclosan (TCS), ibuprofen (IBP) and diclofenac (DCF), achieving high removal rates (64% for DCF, 43% for IBP) and, especially for TCS, with removal rates of 98% [8]. Likewise, HC prepared via HTC using cassava root waste has been employed as adsorbents to remove methyl green and orange in water bodies, obtaining removal efficiencies of up to 60% [9].

The HTC process involves the thermal decomposition of biomass in an aqueous environment under moderate temperatures and high pressures [2,10]. Synthesis conditions, such as temperature and feedstock mass, directly influence the properties of HC and its subsequent its adsorption capacity [11]. Regarding temperature, hydrothermal synthesis is usually performed above 100 °C. However, this parameter may vary depending on the desired properties of the obtained carbonaceous materials. For instance, while some materials may require temperatures as high as 300 °C to achieve a complete reaction, the HTC process is conducted within a range of 150 °C to 300 °C [12].

Methylene blue (MB) is a highly versatile synthetic dye extensively used in scientific and industrial fields. While it serves as a redox indicator in chemistry and an antiseptic in medicine, its widespread application in the textile industry—due to its high color fixation and durability—poses a significant environmental challenge. The discharge of MB-laden effluents into water bodies interferes with photosynthesis and can cause severe toxicological effects on aquatic ecosystems [6]. In this context, HTC has emerged as a promising technology to transform lignocellulosic biomass into HC. This carbon-based material offers a sustainable alternative for wastewater treatment, as its surface properties can be tailored to effectively sequester organic pollutants, like MB through adsorption mechanisms.

This research explores the potential of HC derived from *Rosa rubiginosa* stems, an agricultural residue widely available in Ecuador, as a sustainable and cost-effective adsorbent for removing MB from aqueous solutions. By repurposing waste from the floriculture industry, this work contributes to a circular economy model, effectively turning an environmental burden into a high-value functional material. We systematically investigated how key synthesis variables, specifically temperature and biomass-to-water ratio, shape the physicochemical evolution of the HC and its resulting efficiency in capturing pollutants. Ultimately, establishing these correlations provides a clearer roadmap for designing advanced carbon materials that address the urgent need for eco-friendly water treatment technologies.

## 2. Materials and Methods

### 2.1. Biomass Collection

The rose stem (*Rosa rubiginosa*) residues were collected in a floriculture industry located in Pichincha, Ecuador. The biomass was specifically collected from the sorting area—where stems are graded by quality—following the removal of leaves and flowers. The raw material was then dried for 96 h at 50 °C to remove as much moisture as possible. Finally, the rose stems were ground using an industrial mill to 1 mm fragments, the appropriate size for the hydrothermal synthesis process.

### 2.2. HC Synthesis

To perform the HTC process, a 100 mL hydrothermal synthesis autoclave reactor was used. Next, 5 g of the pretreated feedstock were placed in the reactor with 80 mL of distilled water. The mixture was stirred at 290 rpm for 3 h at room temperature. The reactor was then sealed and heated to 180 °C for 5 h. After cooling to room temperature, the obtained product was washed repeatedly with distilled water until reach a pH of the resulting water between 6 and 7. Finally, the solid carbonaceous material was dried at 60 °C for 24 h.

This HC synthesis process was repeated nine times under different synthesis conditions, varying the raw material mass ratios and temperature, using 80 mL of water in all tests for each case. Table 1 summarizes the synthesis conditions of the HC samples as well as the nomenclature assigned to each sample obtained in this study.

### 2.3. Analysis of HC Production Performance

The mass yield was determined from the ratio of the mass of HC obtained to the initial mass of the raw material (dry biomass) used in the HTC process, using Equation (1):

(1) $$\mathrm{Yield}=\frac{\mathrm{mass}\;\mathrm{of}\;\mathrm{hydrochar}}{\mathrm{mass}\;\mathrm{of}\;\mathrm{dry}\;\mathrm{biomass}\;}\times 100\%$$

### 2.4. Physicochemical Characterization of HC Samples

The point of zero charge (PZC) represents the pH at which the material’s surface net charge is zero, marking its transition from a positively to a negatively charged state. Each sample was determined using the pH drift method [13,14]. Briefly, 50 mL aliquots of 0.01 M NaCl background electrolyte were adjusted to initial pH values (*pH_i_*) ranging from 2.0 to 10.0 using 0.1 M HCl or NaOH. A fixed mass of hydrochar (0.1 g) was added to each solution, and the suspensions were agitated for 24 h at room temperature until equilibrium was reached. The final pH (*pH_f_*) of the supernatant was recorded, and Δ*pH* = *pH_f_* − *pH_i_* was plotted against *pH_i_*. The PZC was identified as the x-intercept where Δ*pH* = 0 [15]. The procedure was repeated for different pH values in order to determine the condition under which the Z potential is zero, thus obtaining the PZC of the material under analysis.

IR analyses of the samples were performed on a Fourier Transform IR Spectrophotometer (Perkin Elmer, Frontier, Waltham, MA, USA) coupled with an ATR cell. To perform this analysis, 20 mg of sample were placed in the ATR-FTIR instrument at room temperature. The baseline was then adjusted, considering air as the background. The IR spectra were collected in the range of 4000 to 550 cm^−1^.

Raman analyses of the samples were performed on a Raman spectrometer (Horiba Scientific, Jobin Yvon Technology, Paris, France), which used a laser source with a wavelength of 532 nm, a potential of 0.2 mW and a spot size of 5 µm [16]. To conduct these analyses, 15 mg of sample were placed on a glass plate and subsequently introduced into the Raman spectrometer, to make a beam of light fall on it. Finally, after a few minutes, the Raman spectra of each HC sample were obtained, which were subsequently treated in order to identify the characteristic bands of this type of carbonaceous materials.

The surface morphology and structural features of the selected HC sample HC5_180) were evaluated using a Scanning Electron Microscope (TESCAN MIRA3 FEG-SEM, Brno, Czech Republic). The analyses were conducted under low vacuum conditions (ranging from 0.1 to 10 Pa). The samples were examined using an acceleration voltage of 5.0 kV, and backscattered electrons were captured using a dedicated detector across various magnifications to observe the surface texture and morphology of the carbonaceous material.

### 2.5. Adsorption Tests

All HC synthesis procedures and methylene blue (MB) adsorption experiments were performed in triplicate. For MB adsorption tests, 25 mg of each HC sample was weighed and mixed with 80 mL of the dye solution, followed by a constant stirring at 150 rpm for 24 h. The adsorption experiments were conducted for the nine HC samples, using different MB initial concentrations (125 mg/L, 100 mg/L, 75 mg/L, 50 mg/L, 25 mg/L, 15 mg/L, 10 mg/L, 5 mg/L and 2.5 mg/L). The initial pH of the MB solutions was adjusted to neutral conditions (pH ≈ 7.0) prior to contacting the HC samples.

The final MB concentration of all solutions was measured to determine the dye removal from each carbonaceous material, in order to identify the optimal HTC conditions. The residual MB concentration in the supernatant solutions after the adsorption tests was quantified using a UV-Vis spectrophotometer (Boeco, S-200, Hamburg, Germany) by measuring the absorbance at the maximum absorption wavelength (*λ_max_* of 664 nm). A calibration curve was established using standard MB aqueous solutions within a concentration range of 0.5 to 8 mg/L (R^2^ = 0.9972). When sample concentrations exceeded the linear working range of the calibration curve, appropriate dilutions with distilled water were performed prior to analysis. The residual MB concentration was determined directly from the linear regression equation of the calibration curve, and the dye removal efficiency and adsorption capacity were subsequently calculated.

#### 2.5.1. Adsorption Kinetics

To determine the kinetics of the MB adsorption onto HC, batch adsorption tests were conducted at various times intervals using 25 mg of HC in 80 mL of dye solution with an initial concentration of 2.5 mg/L. The time of each test started from 0 min and increased until verifying that the adsorption had reached equilibrium. In addition, adsorption isotherms were generated from the equilibrium data, in order to identify the adsorption mechanism for each material, fitting the results to the adsorption models proposed by Langmuir and Freundlich [17,18].

#### 2.5.2. Langmuir and Freundlich Isotherms

The Langmuir isotherm is a fundamental model used to describe the relationship between the adsorbate concentration in the liquid phase and the amount adsorbed on the adsorbent surface under equilibrium conditions [19]. This model is based on certain assumptions, such as monolayer adsorption onto the surface and the presence of energetically equivalent, homogeneous active sites.

To find the Langmuir isotherm, the final concentration data from all the adsorption tests performed were used. First, the “q” value was found using the following equation:

(2) $$q=\frac{\left(\mathrm{Initial}\;\mathrm{concentration}-\mathrm{Final}\;\mathrm{concentration}\right)\times \mathrm{volume}\;\mathrm{of}\;\mathrm{solution}}{\mathrm{Mass}\;\mathrm{of}\;\mathrm{hydrochar}}$$

where “q” is the amount adsorbed at equilibrium, expressed in mg/g.

The Langmuir isotherm was then plotted using the final concentration vs. final concentration/q data. The same procedure was performed for all HC samples obtained in this work.

On the other hand, Freundlich isotherm model describes the relationship between the concentration of a substance in solution and the amount adsorbed onto a solid surface on heterogeneous systems [13]. To fit the Freundlich model, the equilibrium concentration data from the adsorption tests were used. Specifically, the adsorbed amount (q) was first determined, and the Freundlich parameters were then obtained by plotting log(q) versus log (C_e_), where C_e_ corresponds to the equilibrium concentration.

## 3. Results and Discussion

### 3.1. Effect of Process Parameters on HTC Performance

Nine HC samples (see Table 1) were prepared by HTC using rose stem waste as raw material. For the HC synthesis, the biomass weight ranged from 5 to 10 g, with a constant water volume of 80 mL, at HTC temperatures of 180, 200 and 220 °C. According to Table 2, samples HC5_220, HC7.5_200, HC7.5_220 and HC10_220 reached a mass yield of less than 50%. Meanwhile, samples HC5_180, HC5_200, HC7.5_180, HC10_180 and HC10_200, exhibited a yield percentage greater than 50%. Among them, sample HC10_180 showed the highest yield at 57.3%, which suggests that the feedstock mass and synthesis temperature directly affect the amount of HC produced via HTC. Oliveira et al. [20] studied the hydrothermal synthesis using different raw materials, which were cabbage residues, dry straw, forest wood chips and poultry manure, obtaining yields of 34.1, 49.50, 43.9, and 52.5%, respectively. These results are lower than those obtained in this research, which suggests that the use of rose stem waste can be an appropriate precursor to produce HC under the aforementioned HTC conditions [20].

Temperature directly governs the surface chemistry of the material; higher temperatures promote dehydration, decarboxylation, and the volatilization of oxygenated compounds, thereby reducing both the solid yield and the density of surface functional groups. Meanwhile, varying the biomass-to-water ratio modifies the reaction environment without significantly affecting solid recovery. The specific impact of these structural and chemical changes on methylene blue adsorption performance will be analyzed in detail in the following sections.

### 3.2. Physicochemical Characterization of HC Samples

#### 3.2.1. Determination of PZC

The determination of the point of zero charge (PZC) is crucial for understanding the surface properties and behavior of HC-based materials in various applications. The PZC plays an important role in evaluating the removal capacity of organic dyes from wastewater using rose-stem-derived HC [18]. The PZC refers to the pH at which the surface charge of the material becomes neutral, indicating its transition from positive to negative charge. Several methods can be used to determine the PZC of HC, a common approach being the pH drift or mass titration method, which involves measuring surface charge across a range of initial pH values to identify where the net charge equals zero [19]. The PZC of HC is influenced by several factors, including the presence of functional groups on the surface of the material and the composition of the organic dyes to be removed. Functional groups, such as carboxylic acids and phenolic moieties can contribute to the surface charge and dictate the PZC [21].

The PZC of each of the samples was determined by the pH titration technique, as shown in Figure 1.

As seen in Figure 1, samples HC5_180, HC7.5_180 and HC10_180, exhibited the highest PZC values (5.36, 5.22 and 4.58), whereas samples HC5_200, HC7.5_200 and HC10_200 showed an intermediate PZC value (4.56, 4.38 and 4.39). In contrast, samples HC5_220, HC7.5_220 and HC10_220 recorded the lowest PZC values (4.18, 4.31 and 4.26). This variation is directly related to the synthesis temperature: samples prepared at 180 °C yielded the highest PZC, those synthesized at 200 °C showed intermediate values, and those prepared at 220 °C presented the lowest. The PZC values found in this research are consistent with those reported by Amaringo Villa [22], who analyzed HC samples prepared from rice husk and coconut shell, where their PZC values were 5.40 and 4.61, respectively. Similarly, Pesantes [23] determined the PZC of coffee seeds, in which a value of 5.97 was obtained.

In this context, the PZC of the obtained HC samples exhibits variations that can be attributed to the conditions used during the synthesis process. These conditions directly influence the physicochemical properties of the material, especially the formation and distribution of functional groups on the surface. These functional groups are key determinants of surface behavior and the material’s interaction with different chemical species. To evaluate these surface characteristics, Raman and Fourier-transform infrared (FTIR) spectroscopy analyses were performed, the results of which are presented and discussed below.

#### 3.2.2. Structural Characterization via Raman Spectroscopy

The Raman spectra obtained for the HC samples provide key insights into their structural organization. As shown in Figure 2, two characteristic bands were identified: the D band (~1350 cm^−1^), associated with structural defects such as vacancies, edges, and dislocations in the graphitic lattice; and the G band (~1520–1590 cm^−1^), related to the stretching vibrations of C=C bonds in more ordered graphitic domains [13].

The ratio between these bands, expressed as the intensity ratio I_D_/I_G_, is a widely accepted parameter to estimate the degree of disorder or crystallinity in carbonaceous materials [24]. The I_D_/I_G_ ratio was calculated for all HC samples prepared in this study, obtaining 0.89 for HC10_220 and 1.52 for HC10_180. This suggests that higher carbonization temperatures promote a more ordered structure with lower defect density. This behavior is consistent with previous studies on biomass-derived carbon materials, in which a lower I_D_/I_G_ ratio correlates with enhanced crystallinity as a result of thermally induced reorganization of graphitic layers [25]. Moreover, the lower crystallinity and higher structural disorder observed in samples prepared at milder conditions contribute significantly to the accessibility of the carbon matrix. While Raman spectroscopy specifically evaluates the degree of structural disorder (I_D_/I_G_ ratio) and defect density in the carbon skeleton, the presence of accompanying oxygen-containing functional groups—acting as active adsorption sites—is independently confirmed by infrared spectroscopy [26]. The simultaneous presence of structural defects and surface functionalities facilitates the adsorption of external species, such as dyes.

HTC temperature conditions also influence the chemical evolution of HC. At elevated temperatures, a notable reduction in oxygen-containing functional groups occurs, along with increased compaction of the graphitic layers. Conversely, milder treatments tend to preserve disordered structures rich in defects and heteroatoms [27]. This balance between disorder and functionalization allows for fine-tuning of the physicochemical properties of the material depending on the intended application.

Furthermore, it has been reported that the relative intensity of the Raman bands may depend on the abundance and size of the sp^2^ carbon domains responsible for the corresponding vibrational modes. Therefore, a higher content of disordered or functionalized carbon typically results in an intensified D band, as demonstrated in thermally treated HC derived from sewage sludge [28].

Overall, the Raman analysis confirms that thermal treatment is a key factor governing the structure of HC, influencing both the degree of graphitization and the abundance of surface functional groups. This structural evolution directly impacts the potential performance of HC in environmental remediation applications.

#### 3.2.3. Surface Chemistry Analysis via Infrared Spectroscopy

The IR spectra of all HC samples are shown in Figure 3. This reveals the presence of bands characteristic of the functional groups at the surface of these carbonaceous materials. The IR band discernible at around 1700 cm^−1^ has been attributed to C=O stretching vibrations, suggesting the presence of carbonyl groups in the molecular structure [29]. Moreover, the vibration in the region of 1600 cm^−1^ has been associated with C=C bonds, which reflects the existence of conjugated double bonds, characteristic of aromatic structures [30]. While, the peak near 1200 cm^−1^ denotes stretching vibrations of C-O-C bonds, indicative of the presence of ethers [31]. Likewise, the C-H stretching vibrations observed around 1100 cm^−1^ imply C-H bonds in the molecular composition [13]. Additional signals detected in the range of 1150 to 1175 cm^−1^ have been associated with C-C skeletal vibrations, and oxygenated functionalities is further evidenced by peaks at 1530 and 1340 cm^−1^ [30]. These results align with those reported by Quesada et al. for HC samples derived from sugarcane bagasse, where FTIR spectra exhibited specific vibrations, such as axial deformation of the carbonyl group (C=O) between 1750^−1^, 650 cm^−1^, as well as C=C and C=O aromatic bonds at 1600^−1^, 580 cm^−1^. The presence of carbonyl groups (C=O) identified by the peak at around 1700 cm^−1^ suggests the possibility of potential electrostatic interactions with metal ions and organic compounds [32]. Furthermore, the aromatic groups, evidenced by C=C bond vibrations in the 1600 cm^−1^ region contribute to the adsorption capacity, by facilitating *π* − *π* interactions and other dispersion forces with organic species.

The FTIR results obtained for the HC samples correlate closely with the structural data derived from Raman spectroscopy, providing a comprehensive evaluation of their multi-faceted evolution. Specifically, the surface functional groups identified through infrared analysis (e.g., carbonyl (C=O) and ether (C-O-C) moieties) complement—rather than duplicate—the structural insights reflected by the D and G bands regarding defect density and the organization of the carbon network [30]. Furthermore, the presence of aromatic C=C structures, confirmed by the IR band at 1600 cm^−1^ highlights the development of ordered carbon domains during HTC [30]. In addition, the complementary signals observed in both techniques validate the structural ordering and consistency of the carbon network [33]. Ultimately, the convergence of results from both spectroscopic methods confirms that thermal treatment successfully tailors the surface chemistry and structural organization of HC-based materials.

Based on the information obtained from the characterization, it is possible to identify that the samples with the highest abundance and density of surface functional groups were HC5_180 and HC5_200. This would suggest that these carbonaceous materials may have a greater adsorption capacity. To corroborate this hypothesis, batch adsorption tests were performed across all synthesized HC materials to evaluate how HTC conditions dictate surface chemistry and, consequently, dye removal performance. The corresponding results for MB adsorption are presented and discussed below.

#### 3.2.4. Morphological Analysis via Scanning Electronic Microscopy (SEM)

To evaluate the surface morphology and structural features of the synthesized materials, Scanning Electron Microscopy (SEM) analysis was performed on the representative HC5_180 sample, which achieved the highest production yield and superior MB adsorption performance (which is described in Section 3.3).

As illustrated in Figure 4, the SEM micrograph reveals a relatively compact, fibrous surface morphology characteristic of pristine, non-activated HCs produced under mild hydrothermal carbonization conditions. Unlike activated carbons or materials subjected to high-temperature treatments, the micrograph shows a limited development of internal porous networks, displaying instead a solid, structured carbon matrix derived from the original lignocellulosic tissue of the rose stem.

This morphological observation is consistent with the absence of advanced gas sorption (BET) analyses, reinforcing the premise that non-activated HC-based materials possess restricted internal surface areas and low porosity. Consequently, this structural evidence confirms that the adsorption of cationic dyes onto these materials is not governed by physical entrapment within high-volume pores. Instead, as corroborated by the abundant oxygen-containing functional groups identified via FTIR (e.g., C=O and C-O) and the structural defects indicated by Raman spectroscopy (I_D_/I_G_ ratio), the adsorption mechanism is predominantly driven by chemisorption and electrostatic interactions occurring at accessible active surface sites.

### 3.3. MB Adsorption Tests

#### 3.3.1. MB Removal Efficiency

Adsorption tests were performed using different MB initial concentrations to analyze its MB removal efficiency onto HC and to construct adsorption isotherms for each HC sample. The initial pH of the MB solutions was maintained at neutral conditions (pH ≈ 7.0). Considering that the PZC values of the synthesized HC samples ranged between 4.18 and 5.36 (as determined in Section 3.2.1), the solution pH remained above the PZC (pH > PZC) for all materials. Under these conditions, the surfaces of the HC samples carry a net negative charge, which strongly promotes favorable electrostatic attractions with the cationic MB dye molecules. This confirms that electrostatic interactions play a pivotal role in the adsorption process alongside the active surface functional groups identified by FTIR and Raman analyses. By contacting 25 mg of HC to a MB solution of 2.5 mg/L, considerable MB removal values were achieved, as shown in Table 2.

The highest MB removal percentage (76.3%) was obtained with sample HC5_180. The MB removal results achieved in this work were higher than those reported by Pineda and Guarín [34], who prepared HC from spring onion husks, corn husks, and pea hulls, obtaining MB removal percentages of 6.4%, 36.7%, and 22.8%, respectively. Likewise, Pesántez [23] analyzed the MB adsorption on biochars derived from rose stems and coffee grounds, reporting MB removal percentages of 60.4% and 75.0%, respectively.

The removal results obtained with the carbonaceous materials prepared in this study are consistent with the physicochemical properties identified by IR and Raman spectroscopy. According to these characterization results, sample HC5_180 exhibited higher structural disorder (indicated by its Raman I_D_/I_G_ ratio) combined with a greater abundance of surface functional groups (as confirmed by FTIR analysis). This indicates that a higher concentration of active surface functionalities enables the HC sample to achieve a superior MB adsorption capacity, which is directly reflected in its higher removal percentage.

#### 3.3.2. MB Adsorption Kinetics

Kinetic studies were subsequently conducted to determine the reaction order and evaluate the MB adsorption kinetics onto the HC5_180 sample, which exhibited the highest removal efficiency. The experiments were performed using an initial MB concentration of 2.5 mg/L and an adsorbent dosage of 25 mg HC in a volume of 80 mL, and varying contact times to establish the equilibrium time, which was ultimately set at 24 h for all subsequent adsorption isotherm tests to ensure full saturation. Figure 5 shows the final concentration of MB after adsorption on the HC5_180 sample over time. A gradual decrease in the MB concentration, and therefore, an increase in the amount of MB adsorbed on the HC surface was observed, reaching the equilibrium at 480 min (8 h). This equilibrium point indicates that the adsorption and desorption rates of MB on HC5_180 have equalized, representing a state of dynamic equilibrium in the adsorption process, achieving a MB removal percentage of 76.3%, with an adsorption capacity of 6.08 mg MB/g HC.

Fruehwirth [35] analyzed the adsorption kinetics of MB using HC prepared from wood waste, reaching the equilibrium in a time similar to that reported in this research. However, the dye removal percentage obtained in their work was only 26.04% [36]. This indicates that the HC sample prepared via HTC from rose stems serves as a more efficient adsorbent than those reported in the literature. In turn, rose stems wastes generated in Ecuadorian flower industries may be a promising raw material to obtain hydrochar-based materials with interesting properties for environmental remediation, where the presence of functional groups (analyzed by Raman and IR) is very important to promote its adsorption capacity.

The kinetic data were also evaluated to identify the order and limiting step of the process. To this purpose, the Pseudo-first-order (PFO, Equation (3)) and Pseudo-second-order (PSO, Equation (4)) models were considered:

(3) $$\log(Q_{e}-Q_{t})=\log Q_{e}-\left(\frac{k_{1}}{2.303}\right)t$$

(4) $$\frac{t}{Q_{t}}=\frac{1}{k_{2}Q_{e}^{2}}+\frac{t}{Q_{e}}$$

The kinetic data obtained with sample HC5_180 were adjusted to the aforementioned models, which are shown in Figure 6 and Figure 7. According to these results, the experimental data were better adjusted to the PSO model [13].

In a PSO adsorption systems, the rate-limiting step is predominantly governed by chemisorption mechanisms, which suggests that the rate-limiting step may involve valence forces through sharing or exchange of electrons between the adsorbate and adsorbent [37]. Although kinetic models alone cannot entirely rule out physical contributions, this behavior—when evaluated alongside our spectroscopic findings—points toward a dominant role of chemisorption and surface-site interactions. Thus, the high density of active sites explains why sample HC5_180 achieved superior performance, recording a MB removal efficiency of 76.3% and an experimental adsorption capacity of 6.08 mg of MB/g of HC [38].

#### 3.3.3. MB Adsorption Isotherms

MB removal analyses were performed using HC samples prepared with 5 g of rose stem wastes (HC5) at temperatures of 180, 200, and 220 °C, in order to obtain adsorption isotherms for each carbonaceous material. To understand the MB adsorption mechanism onto each HC5 sample, the obtained isotherms were fitted to the adsorption models proposed by Langmuir and Freundlich. Figure 8 illustrates the non-linear fits for the Langmuir and Freundlich models for HC5 samples, and Table 3 lists the corresponding parameters and residual standard errors (RSE).

As seen in Figure 7, the experimental adsorption data for the three HC5 samples show a good agreement with the Langmuir model. This behavior is mathematically described by an apparent monolayer saturation capacity within the evaluated concentration range, further supported by the lower RSE values compared to the Freundlich isotherm. Although biomass-derived HCs are inherently heterogeneous, the mathematical applicability of the Langmuir model serves here as an empirical estimation of maximum adsorption capacity, reflecting adsorption occurring at energetically comparable accessible sites under these milder synthesis conditions.

The preferential fit to the Langmuir model observed in this study is consistent with previous reports for pristine biomass-derived biochars produced under relatively mild pyrolysis conditions. Subratti et al. [39] reported that biochar derived from *Cedrela odorata* and pyrolyzed at 400 °C exhibited equilibrium behavior consistent with the Langmuir isotherm, which was associated with a relatively homogeneous distribution of adsorption sites and a maximum adsorption capacity of 158.8 mg/g. Likewise, Chafi et al. [31] found that biochar produced from exhausted olive pomace at 400 °C for 1 h was accurately described by the Langmuir model (R^2^ = 0.999), suggesting predominantly monolayer adsorption and strong adsorbate–adsorbent affinity. These findings support the interpretation that the Langmuir behavior observed here is compatible with adsorption occurring predominantly at energetically comparable accessible sites, although the intrinsic heterogeneity of biomass-derived carbon surfaces should not be disregarded.

The FTIR and Raman analyses reveal that the HC5 materials hold onto a rich profile of oxygenated functional groups like C=O and C-O, alongside a significant density of structural defects indicated by I_D_/I_G_ ratios dropping from 1.73 down to 1.31. These specific surface sites can act as the primary anchoring points for electrostatic attraction with the cationic MB molecules. Among the carbonaceous materials prepared in this work, the HC5_180 sample stands out by achieving the highest overall MB adsorption capacity, boasting an experimental equilibrium capacity of 68.51 mg MB/g HC and a theoretical Langmuir maximum adsorption capacity (q_m_) of 85.86 mg MB/g HC (it should be noted that this equilibrium capacity differs from the value obtained in the kinetic study (~6.08 mg MB/g HC), as both assays were carried out at different initial dye concentrations). When compared with other non-activated, raw HC-based materials reported in the literature, where removal capacities usually hover between 30 and 80 mg/g, this material demonstrates a very competitive performance. On the other hand, a considerable effect of the synthesis temperature on the MB adsorption capacity obtained for the HC5 samples has been observed. The samples prepared at 200 °C and 220 °C display a gradual drop in their Langmuir maximum MB adsorption capacities, falling to 80.32 mg/g and 77.27 mg/g, respectively. This decrease demonstrates that the increase in the HTC temperature can degrade oxygenated groups and mitigates the formation of structural defects, reducing the affinity of HC for the dye.

Likewise, an analysis of the adsorption isotherms obtained for the samples prepared with 7.5 g of residual biomass and 80 mL of water at temperatures of 180, 200 and 220 °C was performed. The adsorption isotherms of the mentioned samples, along with their fitting to the Langmuir and Freundlich models, are shown in Figure 9. 

As the initial biomass mass increases to 7.5 g, the adsorption behavior begins to deviate from the uniform monolayer pattern observed in the HC5 samples, which indicate an emerging surface complexity. The HC7.5_180 sample showed a better fit to the Langmuir model with a theoretical q_m_ value of 77.92 mg/g, demonstrating that mild temperatures (e.g., 200 °C) moderate temperatures do not degrade HC active sites, even at higher biomass-loading-to-water-volume ratios. This trend is consistent with prior literature on similar carbonaceous materials, which highlights that moderate synthesis conditions can preserve surface functional groups and favorable pore structures that contribute to adsorption. Mittal et al. [40], for instance, reported Langmuir-type homogeneous monolayer adsorption for a carbonaceous adsorbent synthesized through low-temperature hydrothermal carbonization at 453 K. Similarly, Chafi et al. [31] found that biochar produced at 400 °C for 1 h retained abundant surface functional groups and exhibited equilibrium behavior accurately described by the Langmuir model (R^2^ = 0.999), indicating relatively homogeneous adsorption energies. Conversely, Liao et al. [41] demonstrated that increasing activation severity progressively altered the carbon structure, while excessively harsh conditions promoted the decomposition of surface functional groups and the deterioration of microporous features. These observations suggest that the Langmuir behavior observed in the present material may be associated with the preservation of chemically active and energetically comparable adsorption sites under relatively mild synthesis conditions, rather than with surface homogeneity arising solely from extensive carbonization or activation. 

As the temperature increases, mass interactions are modified, triggering a transition in the dye adsorption model. Thus, sample HC7.5_200 exhibited a dual or transitional character; although its RSE gave a slight numerical edge to the Freundlich model (2.3299 versus for Langmuir, reflecting a very similar fit for both models), which suggests the onset of surface heterogeneity, a situation in which neither mechanism completely predominates. This behavior may reflect the onset of greater surface heterogeneity, with adsorption occurring over sites displaying a broader distribution of adsorption energies, which is consistent with the fundamental assumptions of the Langmuir and Freundlich isotherms. Therefore, the comparable performance of both models suggests that the system may exhibit an intermediate adsorption behavior, in which relatively homogeneous adsorption domains coexist with increasingly heterogeneous sites rather than either mechanism being exclusively dominant [17,42].

The aforementioned trend changed by the time the system reached 220 °C. This has been reflected in the adsorption data obtained with the HC7.5_220 sample, where its mechanism shifts clearly toward the Freundlich model, supported by a lower RSE of 1.0264 compared to Langmuir’s 2.6163, with an experimental and theoretical maximum capacity of 50.13 and 64.65 mg/g, respectively. This transition is consistent with the physicochemical characterization, which demonstrated that higher synthesis temperatures and denser packing resulting from the higher biomass content, promote the structural disorder and the secondary char precipitation. These phenomena generate multi-energy sites and a broader distribution of active adsorption sites rather than uniform energetic surfaces, aligning with established reports where heterogeneous carbon structures favor the Freundlich isotherm. For instance, Ščasná et al. [43] reported predominantly Freundlich-type fitting for carbonaceous materials derived from *Paulownia tomentosa*, attributing this behavior to the presence of heterogeneous adsorption sites. Similarly, Paluch et al. [44] found that MB adsorption onto biomass-derived biocarbon was best described by the Freundlich isotherm, suggesting multilayer adsorption on heterogeneous surfaces. These observations support the increasingly comparable contribution of the Freundlich model observed for HC7.5 and suggest a progressive departure from a uniformly energetic adsorption surface toward a broader distribution of adsorption-site energies.

Furthermore, when comparing the experimental adsorption capacities of HC7.5 samples (ranging from 50 to 60 mg/g at equilibrium) with other pristine HCs reported in the literature, the values remain well within the typical performance windows for inactivated biomass-derived adsorbents applied to MB removal. Kozyatnyk et al. [45] demonstrated that inactivated microalgae-derived hydrochars were capable of MB adsorption despite their relatively low surface areas and, importantly, that hydrochars produced at lower HTC temperatures generally exhibited the highest adsorption capacities. Their results further showed that increasing HTC temperature from 180 to 260 °C increased the surface area from 8.5 to 43.6 m^2^/g without producing a corresponding improvement in MB uptake. This behavior was accompanied by changes in surface functionality and carbonization degree, demonstrating that adsorption performance depends not only on pore development but also on the chemical characteristics of the available adsorption sites. Therefore, the MB adsorption capacities obtained for HC7.5 samples indicate competitive MB adsorption for a non-activated HC and reinforce the interpretation that an appropriate balance between surface functionality, pore accessibility, and adsorption-site heterogeneity governs its adsorption behavior.

On the other hand, Figure 10 shows the MB adsorption isotherms obtained on HC samples prepared with an initial mass of 10 g at different temperatures (HC10), as well as their respective fits to the models proposed by Langmuir and Freundlich. As the initial biomass loading increased to 10 g, the adsorption behavior shifted more toward energetic heterogeneity. As seen in Figure 9 and Table 3, all the HC10 samples exhibited a better fit with the Freundlich isotherm, suggesting that the adsorption is not limited to a single molecular layer. Even the sample synthesized at the lowest temperature (HC10_180) showed a better fit to the Freundlich model, yielding a lower residual standard error (RSE of 2.3987) compared to Langmuir (2.4523), along with a theoretical Langmuir capacity and a maximum experimental MB adsorption capacity of 71.72 mg/g and 57.76 mg/g, respectively. This fact displays that a higher biomass-loading-to-water-volume ratio is capable of generating complex microenvironments even at temperatures of 220 °C, where the condensation of intermediates and the formation of secondary char begin to compete with a uniform distribution of active sites.

The MB adsorption behavior onto the HC10 samples has been correlated with their spectroscopic data. The Raman and FTIR spectra suggest that a greater biomass packing density alters the HC structure to a greater extent and broadens the distribution of oxygenated functional groups. Although HC10_180 retains a significant defect density (I_D_/I_G_ index around 1.52), the saturated reaction medium favors the formation of sites with multiple energy levels. This trend became even more pronounced as the HTC synthesis temperature increased to 200 °C and 220 °C. Data for MB adsorption onto HC10_200 and HC10_220 samples exhibited a better fit to a Freundlich isotherm, supported by significantly lower RSE values (1.4961 and 1.7576, respectively) relative to Langmuir. This behavior indicates that high-temperature HTC conditions, combined with a high biomass load, can strip away specific uniform active sites, resulting in heterogeneous surfaces characterized by an exponential energy distribution rather than monolayer saturation. In terms of MB adsorption performance, the experimental MB adsorption capacities and theoretical parameters (q_exp_ values dropping to 50.79 mg/g and 46.41 mg/g for 200 °C and 220 °C) followed a downward trend, which has been associated with the thermal carbonization and the loss of polar functional groups. When evaluated against broader literature reports for inactivated, biomass-derived HCs, the adsorption capacities obtained with the HC materials of the present study remain within a competitive range. More importantly, the observed decrease in adsorption performance at higher synthesis severity is consistent with previous findings showing that increasing HTC temperature and residence time does not necessarily enhance sorption despite promoting pore development. For example, Kozyatnyk et al. [45] studied the effect of temperature of HTC synthesis (from 180 to 260 °C) of microalgae-derived HCs on their MB adsorption capacity, obtaining the highest dye uptake (28.9 mg/g) with the HC prepared under the mildest conditions investigated (180 °C, 2 h), compared with only 1.5–1.9 mg/g obtained with the HC prepared at 260 °C for 6–8 h. This decline was accompanied by progressive changes in surface chemistry, including the weakening of oxygen-containing functionalities and increasing aromatic character. Likewise, Guaya et al. [29] showed that a pristine hydrochar produced under relatively mild HTC conditions (200 °C, 3 h) retained oxygen-containing surface functionalities and achieved an adsorption capacity of 14.94 mg/g. Taken together, these observations support the interpretation that increasing synthesis severity beyond the conditions that provide an appropriate balance between surface functionality and carbonization may alter the distribution and energetic diversity of adsorption sites.

In summary, the comprehensive analysis of the nine HC samples reveals how synthesis parameters determine adsorption performance through structural and chemical modifications, using MB as the test molecule. As demonstrated by the experimental adsorption data (q_exp_), maximum MB removal was achieved with the HC prepared at the lowest temperature (180 °C) and with the biomass loading (5 g), reaching an adsorption capacity of 68.51 mg/g. The spectroscopic findings from FTIR and Raman analyses have provided the physicochemical explanation for this behavior. Mild hydrothermal conditions and low biomass-loading-to-water-volume ratios can generate a high density of oxygenated functional groups (e.g., C=O and C-O) and structural defects (reflected in high I_D_/I_G_ indices), which act as primary active sites that strongly anchor cationic dye molecules via electrostatic interactions. Consequently, these materials exhibited surface homogeneity and uniform site energy distributions, which favored a better fit to the Langmuir isotherm model. Meanwhile, raising the synthesis temperature towards 220 °C and increasing the biomass load to 10 g may accelerate carbonization, structural condensation, and secondary char precipitation. This fact strips away polar functional groups and smooths out defect sites, leading to a steady decrease in both experimental MB experimental adsorption capacities (dropping down to a minimum of 46.41 mg/g for HC10_220). Simultaneously, these more severe, denser and saturated reaction media can promote a multiplicity of energetic sites and structural disorder, causing the adsorption mechanism to naturally shift toward the Freundlich model. Therefore, this correlation proves that surface chemistry and site availability—rather than physical porosity alone—dictate the adsorption dynamics of these pristine HCs, offering a coherent framework for the tailored design of biomass-derived carbon materials.

## 4. Conclusions

This study successfully demonstrated the potential of converting *Rosa rubiginosa* stems, a significant agricultural by-product of the Ecuadorian floriculture industry, into value-added products like high-performance HC obtained via HTC. The results indicate that the synthesis temperature and the biomass-to-water ratio play a pivotal role in defining the material’s final properties. Specifically, the HC produced at 180 °C, using an initial mass of 5 g in 80 mL of water, exhibited the most favorable characteristics for contaminant removal. 

The structural and chemical evolution of the HC was confirmed through IR and Raman spectroscopies, which revealed the retention of critical oxygenated functional groups and a specific degree of structural disorder essential for effective adsorption. Furthermore, the evaluation of the PZC provided key insights into the surface charge dynamics, explaining the favorable electrostatic interactions with the MB molecule. The adsorption performance of the prepared HCs has been primarily attributed to surface chemical interactions, as evidenced by FTIR, alongside the morphological features observed in the SEM micrographs of sample HC5_180. While textural properties such as porosity and surface area would require further dedicated analysis (e.g., BET measurements), the current chemical and morphological evidence strongly suggests that surface-functional interactions play a dominant role in MB removal. These findings underscore the feasibility of a circular economy approach, where floral waste is transformed from an environmental liability into a strategic resource for aqueous remediation. Ultimately, this work provides a scalable technical foundation for developing sustainable water treatment technologies, offering an eco-friendly strategy to mitigate the impact of synthetic dyes through advanced, eco-friendly carbon materials.

## Figures and Tables

**Figure 1:**
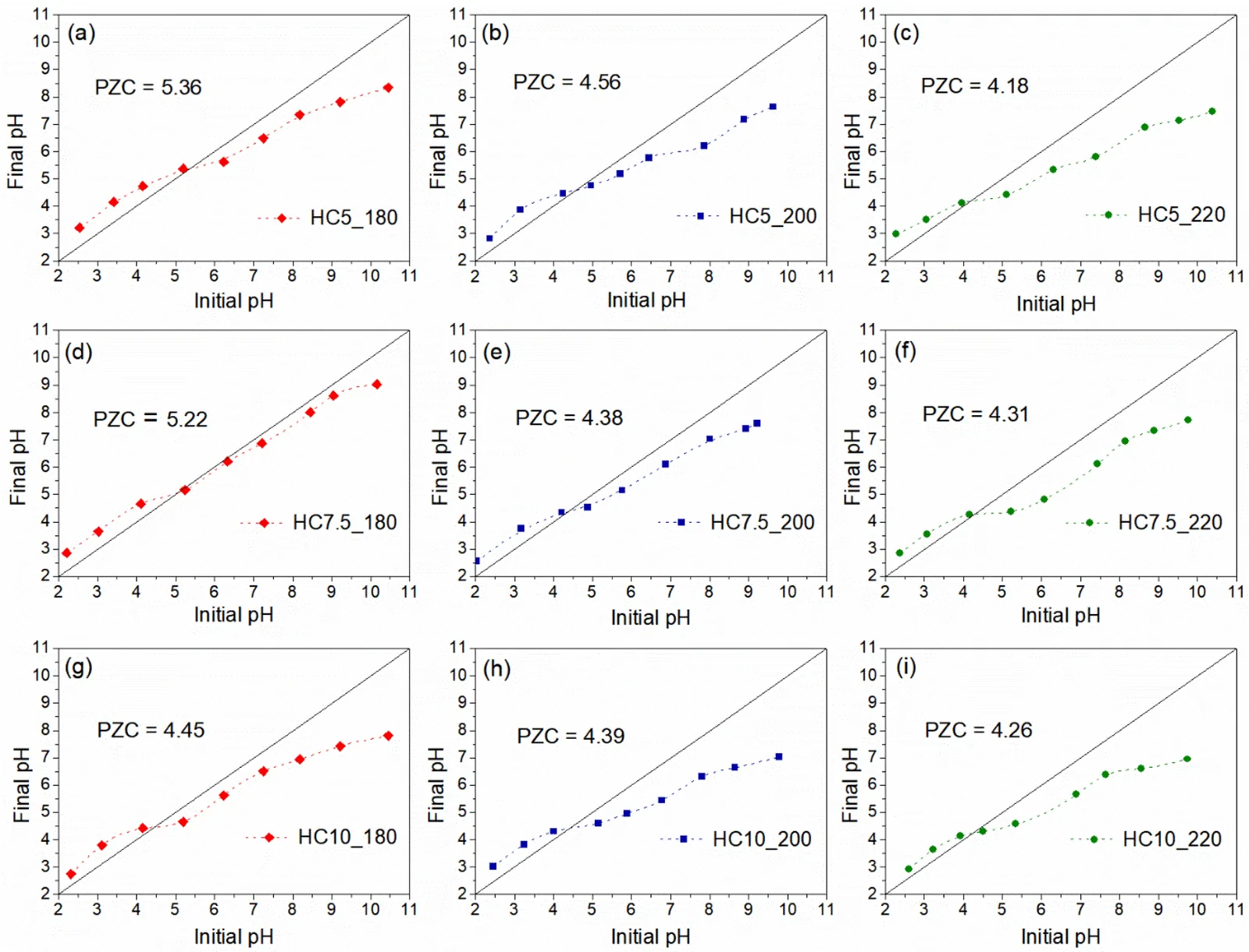
PZC values of HC samples.

**Figure 2:**
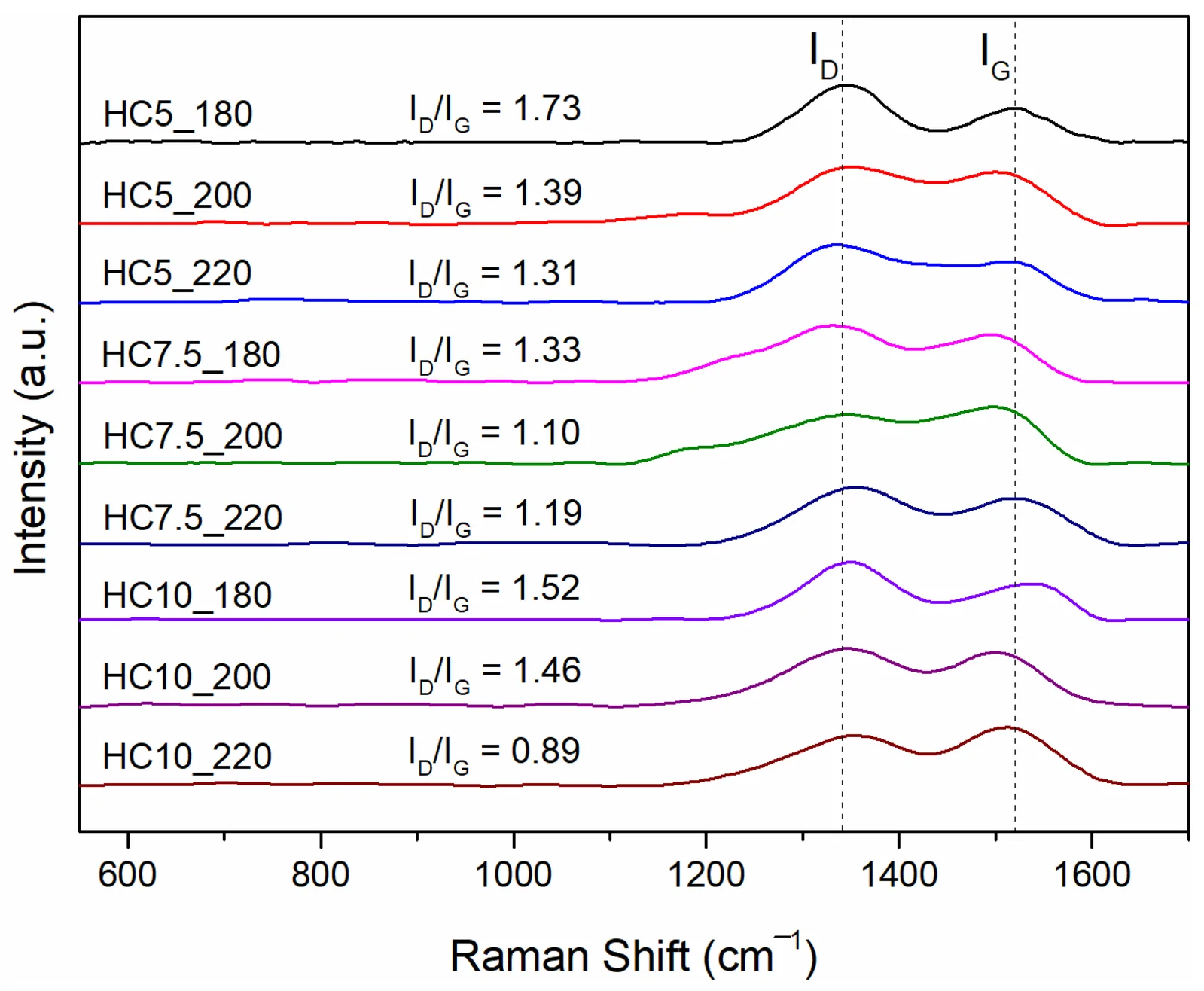
Raman spectra and crystallinity index for each HC samples.

**Figure 3:**
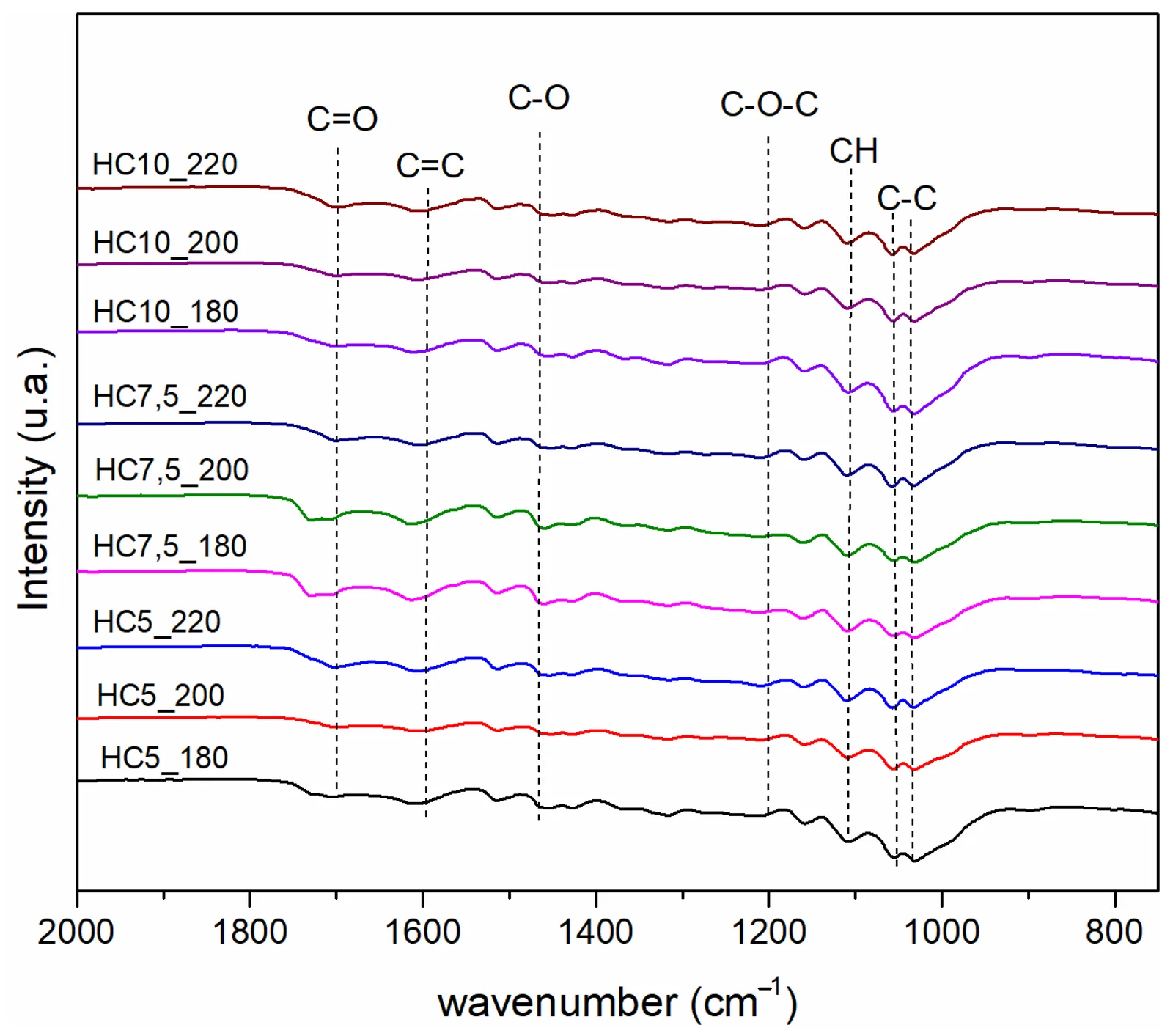
IR spectra of each HC sample.

**Figure 4:**
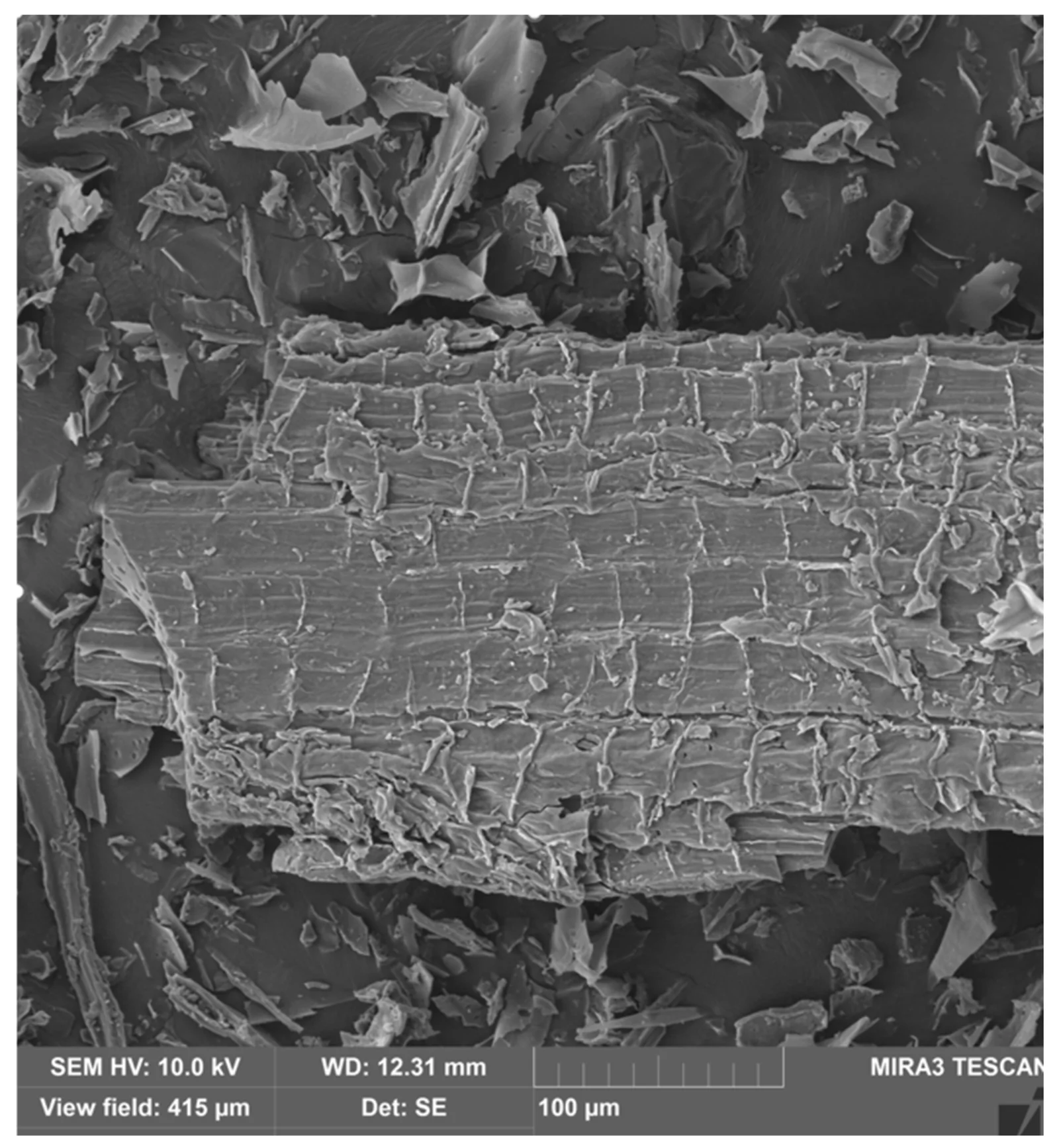
SEM image of HC5_180 sample.

**Figure 5:**
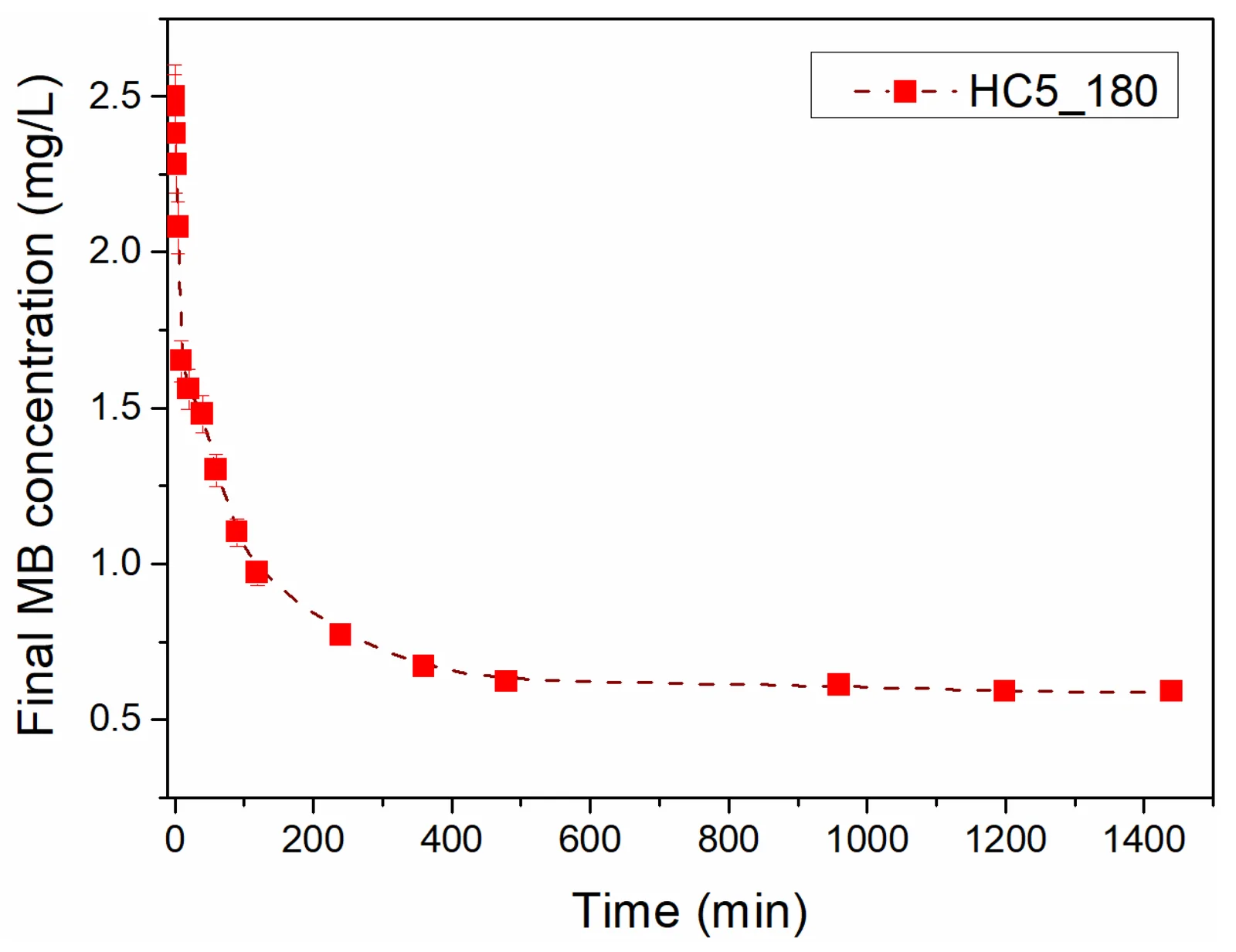
MB adsorption kinetics performed with an initial concentration of 2.5 mg/L using the sample HC5_180.

**Figure 6:**
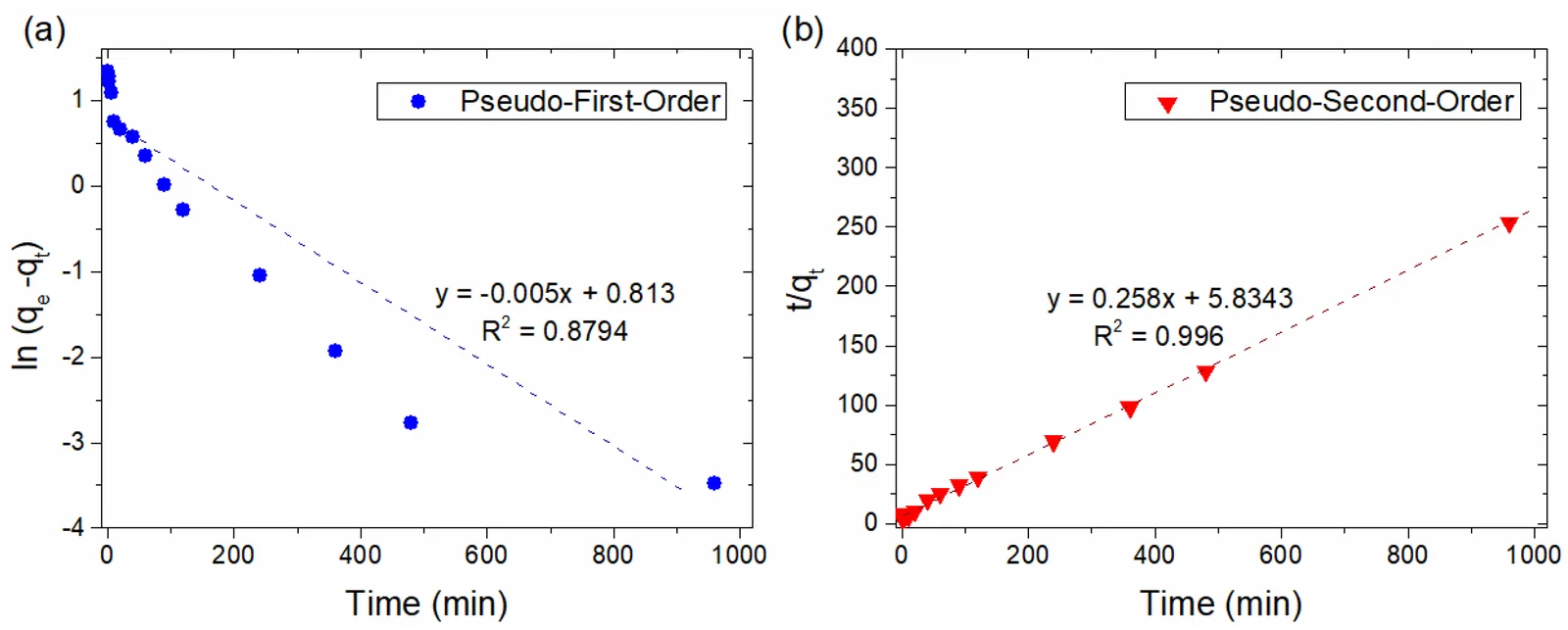
Linearization and fitting of MB absorption data on HC5_180 for PFO (**a**) and PSO (**b**) models.

**Figure 7:**
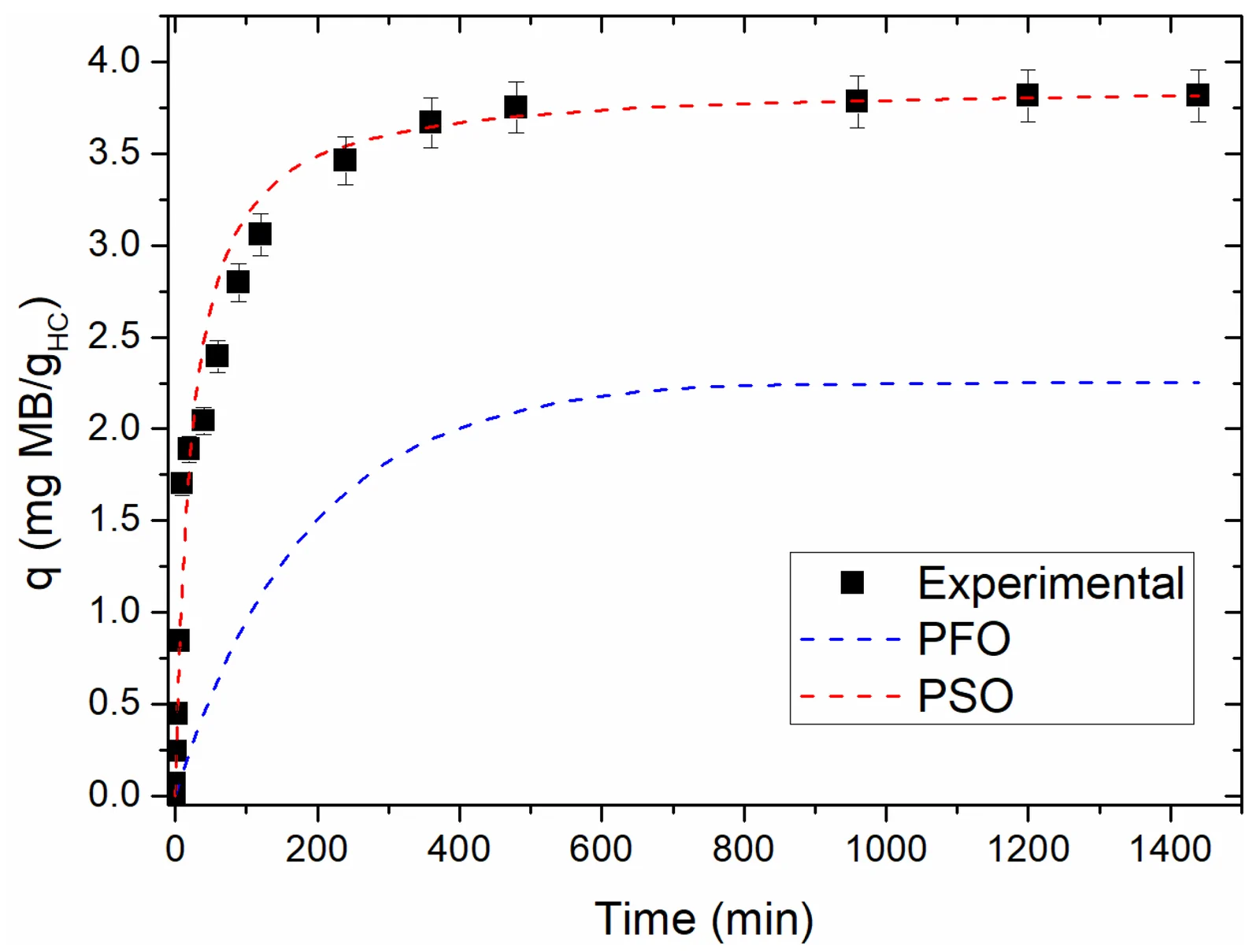
Kinetic data of MB adsorption on HC5_180 sample fitted to PFO and PSO models.

**Figure 8:**
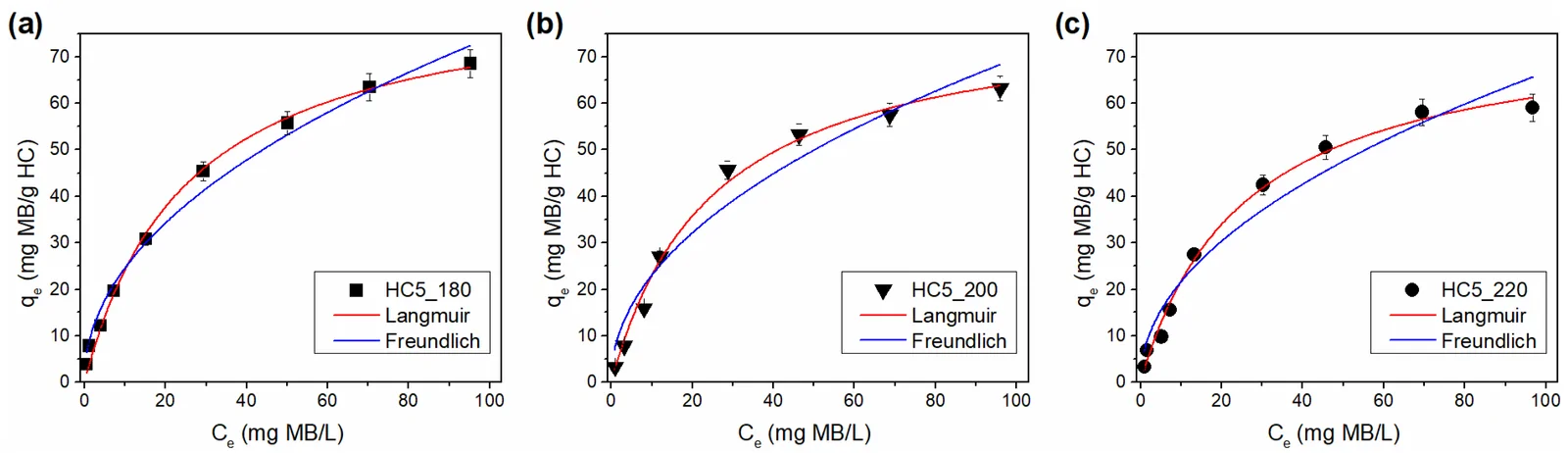
Adsorption isotherms of MB onto HCs synthesized from an initial biomass mass of 5 g at (**a**) 180 °C, (**b**) 200 °C, and (**c**) 220 °C. The red and blue lines represent the fits to the Langmuir and Freundlich models, respectively.

**Figure 9:**
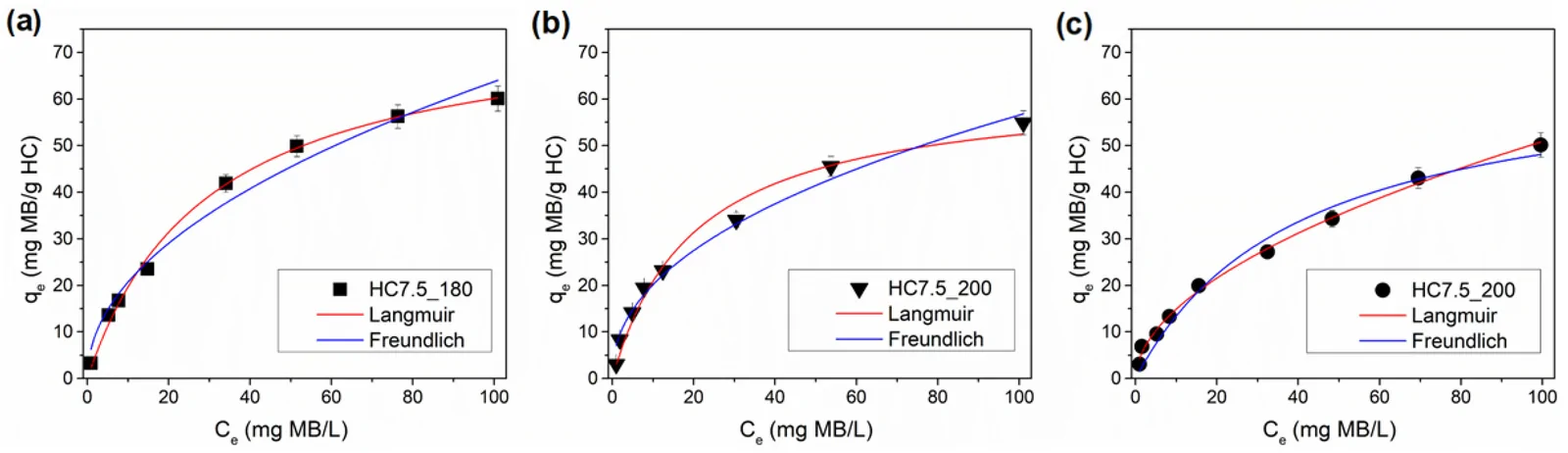
Adsorption isotherms of MB onto HCs synthesized from an initial biomass mass of 7.5 g at (**a**) 180 °C, (**b**) 200 °C, and (**c**) 220 °C. The red and blue lines represent the fits to the Langmuir and Freundlich models, respectively.

**Figure 10:**
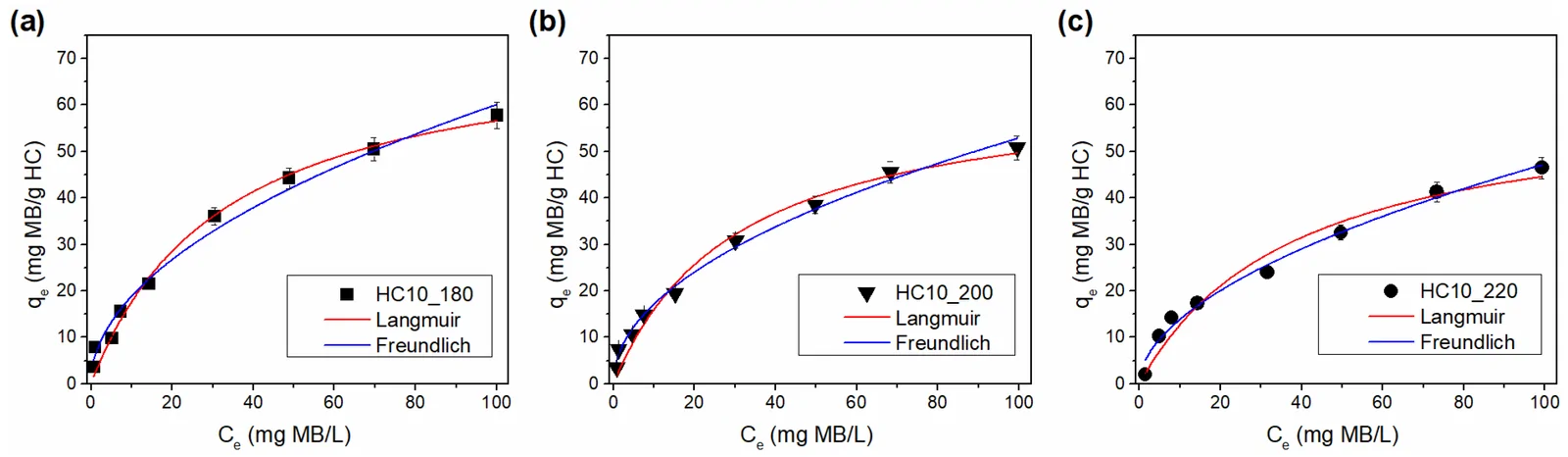
Adsorption isotherms of MB onto HCs synthesized from an initial biomass mass of 10 g at (**a**) 180 °C, (**b**) 200 °C, and (**c**) 220 °C. The red and blue lines represent the fits to the Langmuir and Freundlich models, respectively.

**Table 1:** Nomenclature, synthesis conditions and yield production for each HC sample obtained in this work.

Sample	Nomenclature	Initial Rose Stems Mass (g)	HTC Temperature (°C)	Obtained HC Mass (g)	HC Production Yield (%)
1	HC5_180	5.0	180	2.786	55.72
2	HC5_200	5.0	200	2.697	53.94
3	HC5_220	5.0	220	2.157	43.14
4	HC7.5_180	7.5	180	4.176	55.68
5	HC7.5_200	7.5	200	3.740	49.86
6	HC7.5_220	7.5	220	3.365	44.86
7	HC10_180	10.0	180	5.730	57.30
8	HC10_200	10.0	200	5.382	53.82
9	HC10_220	10.0	220	4.853	48.53

**Table 2:** MB removal percentage achieved with each HC sample.

Sample	Nomenclature	MB Removal Efficiency (%)
1	HC5_180	76.3
2	HC5_200	66.3
3	HC5_220	65.1
4	HC7.5_180	65.7
5	HC7.5_200	61.0
6	HC7.5_220	61.8
7	HC10_180	73.0
8	HC10_200	70.3
9	HC10_220	40.0

**Table 3:** Langmuir and Freundlich isotherm parameters obtained for each HC sample prepared under different synthesis conditions.

Sample	q_exp_ (mg/g)	Langmuir Isotherm Parameters			Freundlich Isotherm Parameters		
		K_L_ (L/mg)	q_m_ (mg/g)	RSE	n	K_F_ (mg/g)	RSE
HC5_180	68.51	0.0392	85.86	1.9861	2.09	8.15	2.9951
HC5_200	63.25	0.0401	80.32	2.0742	2.08	7.62	5.3990
HC5_220	59.04	0.0392	77.27	1.9629	2.04	7.02	4.8303
HC7.5_180	60.10	0.0338	77.92	1.3754	2.05	6.72	3.2377
HC7.5_200	54.89	0.04924	63.31	2.5395	2.23	7.21	2.3299
HC7.5_220	50.13	0.0282	64.65	2.6163	1.89	4.37	1.0264
HC10_180	57.76	0.0307	71.72	2.4523	1.99	5.91	2.3987
HC10_200	50.79	0.0328	64.69	2.6004	2.05	5.57	1.4961
HC10_220	46.41	0.0259	61.79	2.8708	1.88	4.09	1.7576

## Data Availability

The data supporting this study’s findings are available from the corresponding author (C.N.-C.) upon reasonable request.
